# S54. THE ROLE OF THE CLINICAL PHARMACIST IN DRUG EDUCATION FOR INCREASING COMPLIANCE WITH DRUG THERAPY IN THE PERIOD OF DISCHARGE WITH THE DIAGNOSIS OF SCHIZOPHRENIA SPECTRUM DISORDERS

**DOI:** 10.1093/schbul/sby018.841

**Published:** 2018-04-01

**Authors:** Nadir Yalcin, Ayce Celiker, Seref Can Gurel, Sertac Ak, Mumin Kazim Yazici

**Affiliations:** Hacettepe University

## Abstract

**Background:**

The inability to achieve full compliance with drug treatment during the post-discharge period with exacerbations in the illness in patients with schizophrenia and other psychotic disorders is a major problem for the patients themselves, their families, and the healthcare staff in psychiatry.

**Methods:**

In this prospective study, it was aimed to evaluate whether the written and verbal drug education (drug color and shape, interactions, side effects, etc.) given by the clinical pharmacist during the discharge period had an effect on drug compliance. Between 1st September 2016 and 12th June 2017, 40 patients diagnosed with schizophrenia, schizoaffective disorder, schizotypal personality disorder or acute schizophrenia-like psychotic disorder according to ICD-10 diagnostic criteria who were admitted to Hacettepe University Faculty of Medicine, Department of Psychiatry Inpatient Service, were involved in this study. A number of scales were used to evaluate the severity of illness, drug side effects and drug compliance respectively; PANSS; UKU, SAS, BARS, AIMS; MARS and ROMI. It has been emphasized during discharge to the patients by the clinical pharmacist that how important administering the prescribed medicines regularly and as directed. Six to 8 weeks after discharge, the patients were invited to be reevaluated using the scales applied during admission.

**Results:**

There was a statistically significant increase in compliance with treatment as quantitatively assessed by the MARS after drug education (p<0.001). There was no significant correlation between compliance and gender, age, tobacco/alcohol use or marital status. At the same time, a significant correlation between severity of akathisia obtained through BARS and a decrease in MARS scores representing the level of compliance was observed (r: -0.367; p<0.05). A decrease in the baseline MARS score was related to an increase in the total number of hospitalizations (r: -0.325; p<0.05) and the number of psychotropic drugs used (r: -0.316; p<0.05). When the factors that may affect compliance were examined by multiple regression analysis, akathisia was found to have the highest impact on compliance (ß: -0.389, r2: -0.002, F: 0.750).

**Discussion:**

These results support the literature in terms of the importance of the impact of side effects on compliance. As a result of the study, it was seen that drug counseling services given by clinical pharmacists can effectively be employed in psychiatric care, for the rational use of medicines. It appears that it is necessary to take advantage of drug counseling on drug use and to develop strategies to improve drug compliance in psychiatry.

